# Serogroup C meningococci in Italy in the era of conjugate menC vaccination

**DOI:** 10.1186/1471-2334-9-135

**Published:** 2009-08-22

**Authors:** Paola Stefanelli, Cecilia Fazio, Tonino Sofia, Arianna Neri, Paola Mastrantonio

**Affiliations:** 1 Department of Infectious, Parasitic & Immune-mediated Diseases, Istituto Superiore di Sanità, Rome, Italy

## Abstract

**Background:**

To assess changes in the pattern of Invasive Meningococcal Disease (IMD) in Italy after the introduction of conjugate menC vaccine in the National Vaccine Plan 2005–2007 and to provide information for developing timely and appropriate public health interventions, analyses of microbiological features of isolates and clinical characteristics of patients have been carried out. In Italy, the number of serogroup C meningococci fell progressively following the introduction of the MenC conjugate vaccine, recommended by the Italian Ministry of Health but implemented according to different regional strategies.

**Methods:**

IMD cases from January 2005 through July 2008 reported to the National Meningococcal Surveillance System were considered for this study. Serogrouping and sero/subtyping were performed on 179 serogroup C strains received at the National Reference Laboratory of the Istituto Superiore di Sanità. Antibiotic susceptibility testing was possible for 157 isolates. MLST (Multilocus sequence typing), *por*A VRs (Variable Region) typing, PFGE (Pulsed Field Gel Electrophoresis), VNTR (Variable Number Tandem Repeats) analyses were performed on all C:2a and C:2b meningococci (n = 147), following standard procedures.

**Results:**

In 2005 and 2008, IMD showed an incidence of 0.5 and 0.3 per 100,000 inhabitants, respectively. While the incidence due to serogroup B remained stable, IMD incidence due to serogroup C has decreased since 2006. In particular, the decrease was significant among infants. C:2a and C:2b were the main serotypes, all C:2a strains belonged to ST-11 clonal complex and all C:2b to ST-8/A4. Clinical manifestations and outcome of infections underlined more severe disease caused by C:2a isolates. Two clusters due to C:2a/ST-11 meningococci were reported in the North of Italy in December 2007 and July 2008, respectively, with a high rate of septicaemia and fatal outcome.

**Conclusion:**

Public health surveillance of serogroup C invasive meningococcal disease and microbiological/molecular characterization of the isolates requires particular attention, since the hyper-invasive ST-11 predominantly affected adolescents and young adults for whom meningococcal vaccination was not recommended in the 2005–2007 National Vaccine Plan.

## Background

The epidemiology of meningococcal disease following vaccination campaigns and its evolution over time has been extensively reported [1-5]. In countries where meningococcal vaccines are routinely used, studies on changes in the bacterial population, in terms of capsular switching, and/or molecular characterization, have been carried out in order to assess the eventual impact of vaccination not only on transmission but also on carriage [4].

A new epidemiological pattern of Invasive Meningococcal Disease (IMD) has been observed over the last few years in Italy, a country with a low incidence of the disease (range 0.3–0.5/100,000). In particular, cases due to serogroup C *Neisseria meningitidis *reported to the National Surveillance System at the Istituto Superiore di Sanità fell progressively from 54% (115/212) in 2005 to 36% (53/146) in 2008 http://www.simi.iss.it. This reduction could be explained as a consequence of increasing vaccination coverage especially in those regions (17 of 21) that adopted the 2005–2007 National Vaccine Plan in which the conjugate MenC vaccination was recommended. In particular, all infants in the first year of life and individuals who present with splenic dysfunction or immunodeficiency are considered for vaccination [6]. However, the vaccine coverage is uneven since the health system is decentralised and the Italian regions are entitled to implement different meningococcal vaccination policies according to local epidemiology and priorities. This heterogeneous use of the vaccine together with the occurrence of two outbreaks due to serogroup C meningococci with a high rate of fatal septicaemia [7] led us to assess the genetic characteristics of invasive serogroup C meningococci circulating in the country.

One-hundred and seventy nine serogroup C meningococci collected from invasive disease from January 2005 through July 2008 were characterised using a variety of molecular techniques.

## Methods

### Bacterial strains and phenotypic characterization

Within the National Meningococcal Surveillance System, established in 1994, the NRL received 83% (476/574) of *N. meningitidis *strains isolated by local hospital laboratories throughout the country from January 2005 through July 2008.

All the received strains were subcultured to confirm the serogroup by slide agglutination with commercial antisera (Remel Europe, Ltd, UK). Serotypes and serosubtypes were determined by standard whole-cell ELISA with monoclonal antibodies (purchased from NIBSC, UK).

Susceptibilities to penicillin G, rifampicin, ciprofloxacin and ceftriaxone were determined by Etest method (bioMérieux, Italy), according to the manufacturer's instructions.

The breakpoints were those recommended by the European Monitoring Group for Meningococci (EMGM), [8] In particular, the resistant, intermediate and susceptible breakpoints were: penicillin MIC ≥ 1 mg/L, MIC > 0.06 mg/L to MIC < 1 mg/L, and MIC ≤ 0.06 mg/L; rifampicin MIC ≥ 2 mg/L, MIC = 1 mg/L and ≤ 0.5 mg/l; ciprofloxacin MIC ≥ 0.12 mg/L, MIC = 0.06 mg/L and MIC ≤ 0.03 mg/L. For ceftriaxone, MIC ≤ 0.06 mg/l indicates susceptibility.

### *pen*A gene sequence analysis

*pen*A gene sequence analysis was performed exclusively on penicillin intermediate serogroup C strains (n = 100) to determine specific alleles. DNAs were extracted by using the QIAamp DNA minikit (Qiagen, Germany) according to the manufacturer's instructions. The *pen*A gene was amplified and sequenced using primers and conditions described by Zhang *et al*. [9]; the amplicon of 402 bp, corresponding to the amino acid residues 441 to 574 of the transpeptidase domain of the PBP2 protein, was purified and sequenced to identify the corresponding *pen*A allele using the database available at the http://neisseria.org website [10].

### Molecular analyses

Molecular characterization by MLST (Multilocus sequence typing), *por*A VRs (Variable Region) typing, PFGE (Pulsed Field Gel Electrophoresis), VNTR (Variable Number Tandem Repeats) analyses, were performed on C:2a and C:2b meningococci isolated in the study period (n = 147).

MLST of seven genes (*abc*Z, *adk*, *aro*E, *fum*C, *gdh*, *pdh*C, *pgm*) was performed as described by Maiden et al. [11]. Primers, determination of sequence alleles and designation of sequence types were those described on the MLST website http://neisseria.org/nm/typing/mlstdb/. Variable regions (VR) 1 and 2 were submitted to the *N. meningitidis *PorA variable regions database http://neisseria.org/nm/typing/pora, whereas the VR3 corresponded to those indicated by Mölling et al. [12].

PFGE was performed as already described [13]. Briefly, the bacterial DNAs were digested with 30 U of the restriction endonuclease *Nhe*I (NewEngland, Biolabs, USA) overnight at 37°C.

The CHEF-MAPPER II apparatus (Bio-Rad, USA) was used with the following parameters: voltage of 6 V/cm, pulse time of 0.05s to 30s, run time of 24 h. Lambda ladder PFGE marker (New England, Biolabs, USA) was used as a molecular size standard. The gel was stained with 2 mg/mL ethidium bromide, photographed under UV light and digitized with Gel Doc 2000 system apparatus (Bio-Rad, USA). The method applied for VNTR has been reported elsewhere [14]. In brief, four VNTRs (VNTR01, VNTR02, VNTR06, VNTR08) were analyzed for their genetic polymorphisms. The annealing temperatures for the four PCRs were 59°C, 57°C, 63°C and 57°C for VNTR01, -02, -06 and -08, respectively. Five μl of mixed PCR products were electrophoresed on a 2% SeaKem ME agarose gel (Cambrex Bio Science, USA). Gels were stained with ethidium bromide and photographed under UV illumination.

The PFGE and VNTR gel photographs were analyzed using Bionumerics software (v4.61, Applied Maths, Belgium) and dendrograms were constructed by using the Dice coefficient of similarity with the unweighted-pair group method with arithmetic averages. The position tolerance and the optimization were set to 2.5% and 1. 5%, respectively for PFGE; and both were 1.00% for VNTR. PFGE patterns that were <85% similar were considered different.

### Statistical methods

All data were managed and analysed with Epi-Info version 3.3.2. The Chi-square test was used to calculate the differences in serogroup C IMD incidence over time. A p-value of < 0.05 was considered to be statistically significant.

### Ethics

This study did not require approval from an Ethics committee. Data of patients were handled according to the privacy laws in force in Italy. The Istituto Superiore di Sanità (ISS) is the leading technical and scientific public body of the Italian National Health Service and is under the supervision of the Italian Ministry of Employment, Health and Social Policies.

## Results

### Bacterial strains and clinical data of IMD

**Figure 1:**
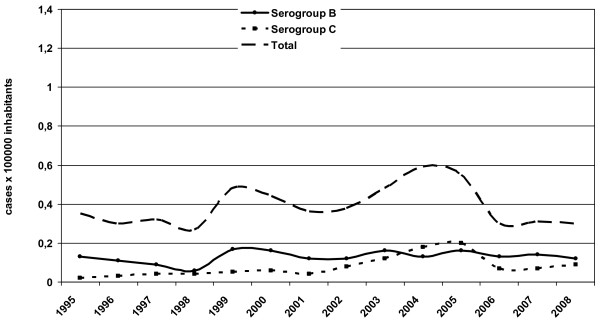
**Annual incidence of serogroup B and C invasive meningococcal disease in Italy, 1995–2008 (cases per 100,000 inhabitants)**.

Trends in IMD incidence in Italy from 1995 to 2008 are shown in Figure 1. The incidence of serogroup B remained stable while the incidence of serogroup C started to decrease in 2005

Table 1 shows serogroup B and C IMD incidence in 2004 and 2007 in different age groups. IMD incidence due to serogroup C decreased significantly (*p *< 0.05) among infants and children aged 0 to 4 years, with an incidence of 0.5 per 100,000 inhabitants in 2007 *vs*. 1.7 in 2004 (Table 1).

**Table 1:** Invasive meningococcal disease incidence in Italy in 2004 and 2007

Age (years)	* Incidence		* Incidence due to serogroup B		* Incidence due to serogroup C	
	**2004**	**2007**	**2004**	**2007**	**2004**	**2007**
**0–4**	4.2	2	0.9	1.1	1.7	0.5 (p < 0.05)
**15–24**	1	0.7	0.3	0.3	0.2	0.2
**25–99**	0.2	0.1	0.05	0.04	0.04	0.02
**All ages**	0.6	0.3	0.1	0.3	0.2	0.06 (p < 0.05)

* reported rates per 100,000 population

From January 2005 through July 2008, 476 meningococci were received by the NRL, of which 179 serogroup C strains from patients with IMD. Serogroup C was predominant in 2005: 54% (97/195), then the proportion decreased to 26% (28/106) in 2006 (p < 0.05) and remained low in the 0–4 year age-group but increased in the other age groups overall: 33% (18/55) in 2007 and 46% (23/50) in the first seven months of 2008.

Analysis of serogroup C serotypes showed a considerable change: C:2a increased from 15% (15/97) in 2005 to 48% (13/27) in 2008, a trend which was inversely paralleled by a decrease of C:2b from 70% (67/97) to 30% (8/27) in the same period, (p < 0.05).

The clinical presentation of invasive meningococcal C disease was reported for 88% (157/179) of cases; outcome was known for 81% (145/179) and underlined more severe diseases caused by C:2a isolates, with an over two-fold increase in septicaemia from 28% (4/14) in 2005 to 70% (9/13) in 2007. Septicaemia cases due to C:2b remained stable: 45% (25/55) and 33% (2/6), respectively Fatal cases due to C:2a meningococci increased from 7% (1/14) to 55% (6/11), p < 0.05.

The serogroup C serosubtypes P1.5, P1.5,2, P1.2 were the most frequently found.

### *pen*A gene analysis

A high proportion of serogroup C strains (65%, 100/157) showed decreased susceptibility to penicillin (PenI), and 82% (82/100) of these were phenotype C:2b. The *pen*A sequence analysis revealed the presence of three main alleles: *pen*A3, and *pen*A9 for C:2a and *pen*A12 for C:2b. All the examined strains were fully susceptible to rifampicin, ciprofloxacin and ceftriaxone.

### Molecular analyses

Table 2 summarizes the molecular characterisation of serogroup C isolates. MLST grouped all C:2a isolates in the ST-11/ET-37 complex and all the C:2b isolates in the ST-8 complex/Cluster A4. Among the 49 C:2a strains examined, 5 PorA genotypes were detected: 5, 2, 36-2; 5-1, 10-8, 36-2; 7-2, 13-1, 36-2; 5, 2-1, 36-2; 5, 10-4, 36-2. Among the 98 C:2b strains, a lower variability was found, in particular 2 PorA genotypes has been detected: 5, 2, 36-2 and 5, 2-1, 36-2.

**Table 2:** Molecular analyses of serogroup C meningococci circulating in Italy from January 2005 through July 2008

Serogroup/serotype	ST/clonal complex	*por*A VR genotypes	PFGE patterns	*pen*A alleles	Total N°
C:2a	ST-11/ET-37	5, 2, 36-2; 5-1, 10-8, 36-2; 7-2, 13-1, 36-2; 5, 2-1, 36-2; 5, 10-4, 36-2	A-L	A3, A9	49
C:2b	ST-8/A4	5, 2, 36-2; 5, 2-1, 36-2	M-N	A12	98

**Figure 2:**
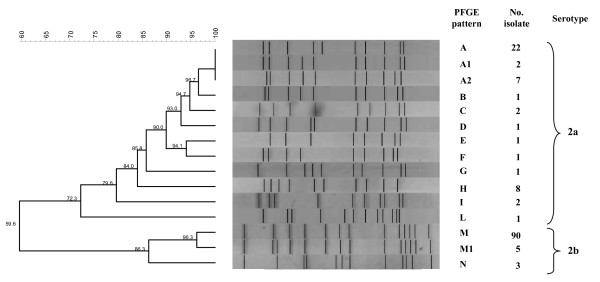
**Dendrogram of similarity obtained with Dice coefficients and arithmetic averages (UPGMA) clustering method using BioNumerics software (version 3.5) showing the relatedness of representative Pulsed Field Gel Electrophoresis patterns of *Nhe *I-digested DNAs of 147 C:2a and C:2b meningococci**. Patterns that were <85% similar (see scale in the upper left corner) were considered different.

Fifteen PFGE patterns were found (Figure 2): twelve among C:2a/ST-11 strains of which three were the most frequent: pattern A (22 isolates), pattern A2 (7 isolates), and pattern H (8 isolates). Patterns from A to G refer to strains with a genetic similarity >85%.

A higher genetic relatedness was found among C:2b/ST-8 strains. Three patterns have been identified: pattern M in 90 strains which differed for only 1 band from pattern M1, found in 5 strains, and from pattern N, found in 3.

**Figure 3:**
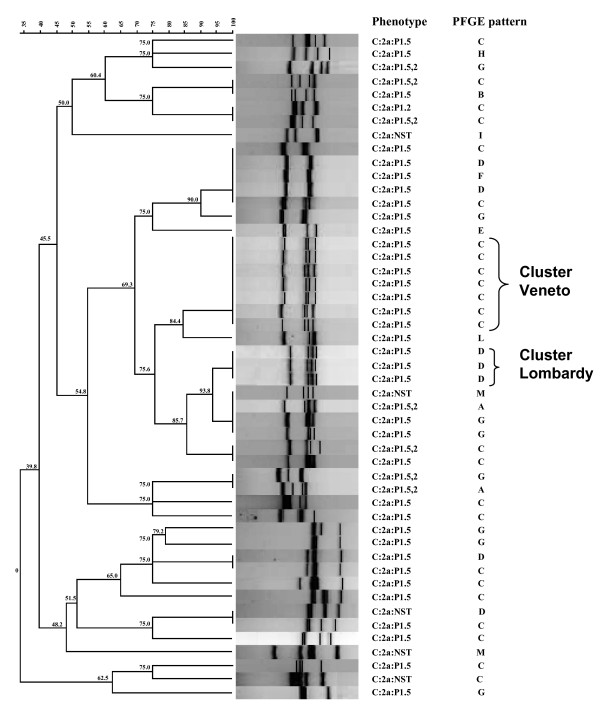
**VNTR patterns among meningococcal C:2a isolates recovered from locally related isolates (Veneto and Lombardy cluster strains) in Italy**. On the right, phenotypes and PFGE patterns are reported.

As already observed in PFGE, the results of the VNTR analysis also showed fewer patterns among C:2b/ST-8 isolates (data not shown) than those observed among C:2a/ST-11 meningococci. In figure 3, the 28 VNTR patterns of the 49 C:2a/ST-11 strains analyzed are shown. VNTR confirmed the similarity observed with the PFGE patterns of the C:2a strains responsible for the two clusters which occurred in December 2007 and in July 2008, respectively, in the North of Italy. In particular, seven isolates belonging to the first cluster (region Veneto) were characterized by the PFGE pattern A, the most frequently found among C:2a/ST-11 strains; whereas, the three isolates belonging to the second cluster (region Lombardy) were characterized by pattern A2. Two different VNTR patterns characterized strains from each cluster and they were different from all the others found in ST-11 complex isolates circulating in Italy in the same period.

## Discussion

Several countries have documented the effectiveness of meningococcal conjugate C vaccines; in particular, vaccination campaigns in the UK, Greece and Spain triggered a rapid decrease in cases of serogroup C disease [3,5,15]. However, there is also evidence that in the absence of vaccination disease incidence may fall as naturally acquired immunity rises [16,17] but this is not a rapid process [2,18].

The serogroup C isolates responsible for numerous epidemics and outbreaks in the United States, Canada and Europe, since the early 1990s, mainly belonged to the ST-11 complex. Despite low carriage rates, ST-11 meningococci continue to be associated with sporadic outbreaks worldwide [4]. These hyper-invasive strains require massive public health investigations and interventions due to the high mortality among cases [19-21].

In Italy, serogroup C meningococcal conjugate vaccine, available since 2002, was recommended in the 2005–2007 National Vaccine Plan; this Plan was adopted in 17 of the 21 Italian regions. Of the 17 Italian regions, nine included vaccination free of charge for all infants, whereas vaccine at reduced cost was available for infants in six additional regions [6]. In particular, all infants in the first year of life and individuals who present with splenic dysfunction or immunodeficiency were considered for vaccination.

Notification of IMD in Italy is mandatory since 1990 and incidence is historically low (range 0.3–0.5 per 100,000 inhabitants). In Europe, as reported by official sources such as the European Union Invasive Bacterial Infections Surveillance Network http://www.euibis.org/documents/2006_meningo.pdf and the European Centre for Disease Control http://ecdc.europa.eu/en/publications/Pages/Surveillance_Reports.aspx, notification rates vary widely between countries, ranging from 0.25 to 4.4 per 100, 000. These numbers reflect real differences in incidence, but also differences between surveillance systems.

In Italy, incidence rates vary and are highest in the North and lowest in the South. Factors such as climate or population density may contribute to these differences. The NRL receives an average of 80% of all strains isolated annually in the country which provides a highly representative pattern of IMD in Italy.

Although the population dynamics of meningococcal disease are characterized by serogroup B and C historically undergoing cyclic patterns, the decrease in serogroup C in a specific age group (infants under 5 years of age) may be associated with menC vaccination. In addition, the majority of serogroup C meningococci circulating during 2008 (data not shown) among non vaccinated people are strains of the hypervirulent C:2a/ST-11 complex (48%), which has replaced the previously predominant C:2b/ST-8 clone (30% in 2005). These strains are hyper-endemic and are responsible for an increased number of fatal cases of septicaemia and for two clusters of cases in adults in the North of Italy [7]. PFGE results showed a higher degree of clonality among C/ST-8 strains compared with C/ST-11 strains, similar to previously published work [22,23]. With regard to the two clusters, strains with identical phenotypes and belonging to ST-11 complex, showed two different profiles by PFGE and VNTR, each unique for its respective cluster and different from all the others found in ST-11 complex isolates circulating in Italy in the same period.

Previous studies demonstrated that conjugate vaccines not only protect vaccinees but also reduce the carriage and the transmission among non vaccinated people [1]. This was observed especially among ST-11 strains after mass vaccination campaigns. In Italy, vaccine coverage is influenced by the different regional policies and in the absence of widespread immunisation among infants, children and adolescents, herd effect among the population is very likely low compared to other European Countries and may have allowed the expansion and spread of specific hyper-virulent clones of meningococci.

## Conclusion

Serogroup C invasive meningococcal disease needs to be carefully monitored by characterizing the isolates since hyper-virulent ST-11 meningococci are currently circulating in adolescents and young adults. This information may be useful for eventual amendments to the current vaccination policies. Moreover, the waning of effectiveness of infant-only scheduled vaccination will be a focal point for future evaluation.

## Competing interests

The authors declare that they have no competing interests.

## Authors' contributions

PS designed the study and wrote the manuscript. CF carried out the phenotypic and genotypic analyses on the strains. TS extracted and analysed microbiological and clinical data from the IMD database. AN carried out the DNA extraction and amplification reactions. PM critically reviewed the study design and manuscript. All authors have read and approve the final manuscript.

## Pre-publication history

The pre-publication history for this paper can be accessed here:

http://www.biomedcentral.com/1471-2334/9/135/prepub
